# Prevalence and correlates of oral antibiotic use in Canada

**DOI:** 10.14745/ccdr.v50i09a04

**Published:** 2024-09-05

**Authors:** Glenys Smith, Anna-Louise Crago, Stephanie Alexandre, Denise Gravel-Tropper, Melissa Isada, Braden Knight, Jami Mackenzie, Jayson Shurgold

**Affiliations:** 1 Antimicrobial Resistance Task Force, Public Health Agency of Canada

**Keywords:** antibiotic, antibiotic use, antibiotic resistance, antimicrobial resistance, Canadian Community Health Survey, Canada

## Abstract

**Background:**

Antimicrobial use (AMU) is a known driver of antimicrobial resistance. Insight into prevalence and correlates of AMU can help identify health inequities and areas for targeted action. To better understand sociodemographic and medical dimensions of AMU in Canada, the Public Health Agency of Canada, in partnership with Statistics Canada, developed a Rapid Response Module questionnaire on self-reported oral antibiotic use, to be administered as part of the 2018 Canadian Community Health Survey (CCHS).

**Objective:**

To provide data on the proportion of people in Canada that self-report the use of antibiotics and sociodemographic and health factors associated with use.

**Methods:**

This cross-sectional study used data from the CCHS, a national survey of 24,176 people with a clustered multi-stage stratified random sampling design. In 2018, an antibiotic use module was administered to CCHS participants.

**Results:**

Among respondents 18 years and older, 26% reported receipt of at least one oral antibiotic over the past year. Several sociodemographic and health factors had higher adjusted odds of receiving an antibiotic prescription, including those aged 18 years compared to aged 48 years (mean), women compared to men, immigrants compared to non-immigrants (excluding Indigenous), current and former smokers compared to those who have never smoked, and those with comorbidities (asthma, chronic obstructive pulmonary disease, arthritis, heart disease, cancer, bowel disorder and urinary incontinence).

**Conclusion:**

Variations in AMU across different key populations and sociodemographic groups highlight the need to improve our understanding of different drivers of AMU and for tailored interventions to reduce inequitable risks of antimicrobial resistance.

## Introduction

Antimicrobial resistance (AMR) is an increasing threat to global health ((1)). In Canada, resistance is increasing for most human pathogens of concern ((2)). Antibiotic use is associated with the development of antibiotic resistance at the individual, community, and country levels, making it imperative to identify and reduce use that is unnecessary or inappropriate ((3,4)). While there are no national-level data, studies in Ontario and Alberta have found that 15.4% and 39.2% of antibiotics were inappropriately prescribed, respectively ((5,6)). For older adults (over the age of 65 years), evidence from Ontario and British Columbia suggests that 50% of antibiotics in the community are prescribed for conditions not requiring antibiotics ((7)).

There is robust evidence of sociodemographic differences in antibiotic use in high-income countries, with a dominant trend of higher use among the elderly, people with underlying medical conditions, women, people with a low income, people with low formal education and various ethnic groups ((8)). This suggests differential drivers of antibiotic use some of which may be linked to health inequities such as disparities in the burden of infection among different population groups or differential rates of inappropriate prescriptions.

While national surveillance of human antimicrobial use (AMU) in Canada reports on the tonnage of antibiotics and number of antibiotic prescriptions dispensed by Canadian pharmacies ((2)), this study provides self-reported data on the proportion of people in Canada reporting use of antibiotics and sociodemographic and health factors associated with AMU. These data are key to elucidating drivers of AMU, developing strategies for community-based antibiotic stewardship and preventing AMR health inequities.

## Methods

### Data source, study design and sample population

This cross-sectional study used data from the Canadian Community Health Survey (CCHS), a voluntary national survey with a clustered multi-stage stratified random sampling design that collects information on health status, determinants of health and healthcare utilization ((9)). There are certain limitations to the sampling methodology, as it excludes those living on reserves or other Indigenous settlements, institutionalized populations (e.g., residents of healthcare facilities, prisons, convents), full-time members of the Canadian Forces, children living in foster care and residents of the remote Québec regions of Nunavik and Terres-Cries-de-la-Baie-James ((9)). Altogether, these exclusions represent less than 3% of the Canadian population aged 12 years and over ((9)).

Along with the core questions of the CCHS, the rapid response component is offered to organizations interested in national estimates on an emerging or specific issue related to the population's health ((9)). To gain further insights into antibiotic use in humans within Canada, the Public Health Agency of Canada, in partnership with Statistics Canada, developed a Rapid Response Module questionnaire on AMU. Between January 2 and June 30, 2018, a nine-question antimicrobial use Rapid Response Module with a focus on antibiotics was administered to 24,176 consenting CCHS participants from all provinces (the territories were excluded). We excluded participants who responded with "don't know", "not stated", or "refused" when asked if they had received antibiotic prescriptions in the past year (n=250), resulting in a final total of 23,926 Canadians aged 18 years and older. Relevant information, including prescribing facility, whether guidance on use was provided, adherence, type of non-adherence, medical reason for prescription and the fate of leftover antibiotics was associated with each outcome. For the complete list of the AMU Rapid Response Module items, please refer to **Appendix**.

### Outcome variable

The outcome for the logistic regression was receipt of one or more outpatient oral antibiotic medication prescription(s) in the 12 months prior to survey administration, regardless of whether the participant filled the prescription.

### Exposure variables

Pre-selected sociodemographic exposure variables were chosen based on clinical plausibility and previous literature. They included age, sex, highest household level of education, household income, smoking status, marital status and specific chronic medical conditions captured in the CCHS ((9)). Body mass index, immigrant/Indigenous status, receipt of previous year influenza vaccination, access to a regular healthcare provider and insurance for prescription medications was also explored. Perceived physical and mental health, as well as perceived stress were also assessed.

### Statistical analysis

Descriptive statistics were used to summarize responses from the AMU Rapid Response Module. Adjusted and unadjusted multivariable logistic regression analyses were performed to evaluate the association between previous year AMU and the pre-selected exposure variables. Age was defined using a five-knot restricted cubic spline ((10)) and all other variables were treated as categorical. Each variable was included in a separate logistic regression model to examine its unadjusted effect on AMU in the previous 12 months. A final model, with all predefined exposure variables, was used to determine which factors maintained their association with AMU in the previous 12 months, adjusting for all other variables. The model included the following variables: sex, age, highest level of education, smoking status, Indigenous status (off-reserve), immigrant status, total household income (in thousands), perceived health, perceived life stress, having asthma, having chronic obstructive pulmonary disease, having arthritis, having high blood pressure, having high blood cholesterol/lipids, having heart disease, ever having been diagnosed with cancer, having a bowel disorder (Crohn's disease, ulcerative colitis, irritable bowel syndrome, incontinence), having urinary incontinence, usual place for immediate care for minor problems, regular provider type, province of residence, marital status, body mass index, type of drinker, level of physical activity, insurance for prescription medications, language most often spoken at home, perceived mental health, having received a seasonal flu shot, having had a stroke, having diabetes, having a mood disorder and having an anxiety disorder. Statistical significance was set at a *p*-value of ≤0.05.

Given the complex sampling strategy of the CCHS, participants had unequal probabilities of being selected for the survey. To account for this, the logistic regression applied sampling weights provided by Statistics Canada to extrapolate the results to the overall Canadian population represented by the CCHS. Bootstrapping weights were used to estimate 95% confidence intervals through a bootstrap variance estimation method (1,000 replications).

All analyses were conducted using SAS Enterprise Guide 7.1 (SAS Institute, Cary, North Carolina, United States). To allow for the proper application of the sampling and bootstrap replicate weights, SAS survey analysis procedures were used.

## Results

Among the CCHS survey respondents 18 years of age or older who completed the 2018 AMU Rapid Response Module (n=23,926, representing a weighted national population of 29,020,553), 26.0% (95% CI: 24.96%–26.99%) reported receipt of at least one oral antibiotic during the previous year (**Table 1**). Of these, 38.2% (95% CI: 36.16%–40.21%) reported receiving more than one prescription. The majority of patients received their antibiotic prescription from community physician clinics (81.8%, 95% CI: 78.19%–85.36%). The reason for prescription was most commonly for infections of the upper respiratory tract (nose, throat or sinus), ear and eye (23.2% combined, 95% CI: 21.41%–25.02%), followed by chest infections (21.5%, 95% CI: 19.41%–23.51%).

**Table 1:** Responses to antimicrobial use questions asked in the Canadian Community Health Survey

Responses	Unweighted		Weighted	
	Frequency	Percent (%)	Frequency	Percent (%)
**Did you receive a prescription for antibiotics in the past 12 months (oral antibiotic)?**				
Yes	6,407	26.78	7,537,172 (7,243,253–7,831,091)	25.97 (24.96–26.99)
Did not fill prescription^a^	61	0.95	49,548 (33,225–65,872)	0.66 (0.44–0.88)
Still taking it	189	2.95	200,614 (153,032–248,195)	2.66 (2.04–3.29)
No	17,519	73.22	21,483,380 (21,189,461–21,777,300)	74.03 (73.01–75.04)
**Did you receive more than one prescription in the past 12 months?**				
Yes	2,541	39.66	2,878,101 (2,691,207–3,064,995)	38.19 (36.16–40.21)
No, just one	3,866	60.34	4,659,071 (4,420,023–4,898,120)	61.81 (59.79–63.84)
**Why were you given a prescription for antibiotics?**				
Chest infection	1,430	21.90	1,617,409 (1,445,932–1,788,885)	21.46 (19.41–23.51)
Ear/nose/throat/sinus/eye infection	1,467	22.90	1,750,049 (1,604,002–1,896,095)	23.22 (21.41–25.02)
Urinary tract infection	978	15.26	1,122,468 (1,002,702–1,242,234)	14.89 (13.39–16.39)
Skin infection	484	7.55	608,859 (502,866–714,851)	8.08 (6.72–9.43)
Gastrointestinal infection	253	3.95	325,678 (259,782–391,573)	4.32 (3.46–5.18)
Other	1,822	28.44	2,112,711 (1,948,239–2,277,183)	28.03 (26.1–29.96)
**Where did you receive the prescription?**				
Walk-in/doctor's office	4,227	65.97	5,243,770 (4,986,062–5,501,478)	69.57 (67.47–71.67)
Outpatient clinic	991	15.47	919,754 (799,370–1,040,139)	12.2 (10.72–13.69)
Inpatient	272	4.25	263,550 (213,118–313,982)	3.5 (2.83–4.17)
Dentist	745	11.63	877,336 (770,540–984,133)	11.64 (10.27–13.01)
Another place	172	2.68	232,762 (170,137–295,387)	3.09 (2.26–3.92)

^a^ Weighted frequencies have limited interpretation due to small response rates

**Figure 1:**
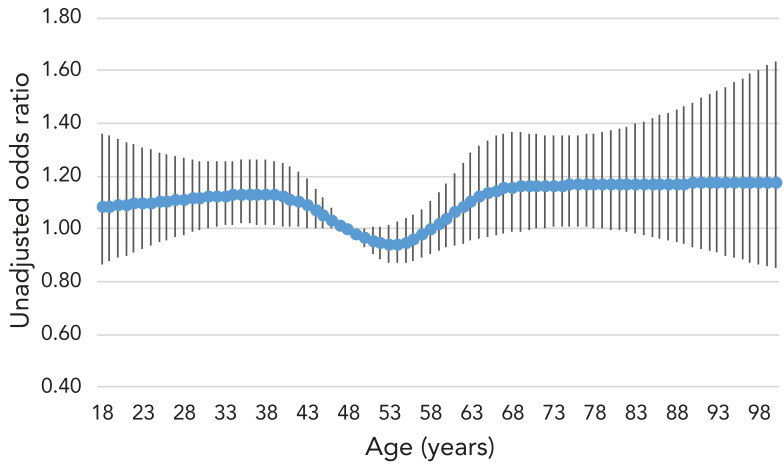
Unadjusted odds ratio for oral antibiotic use in the past 12 months by age

**Figure 2:**
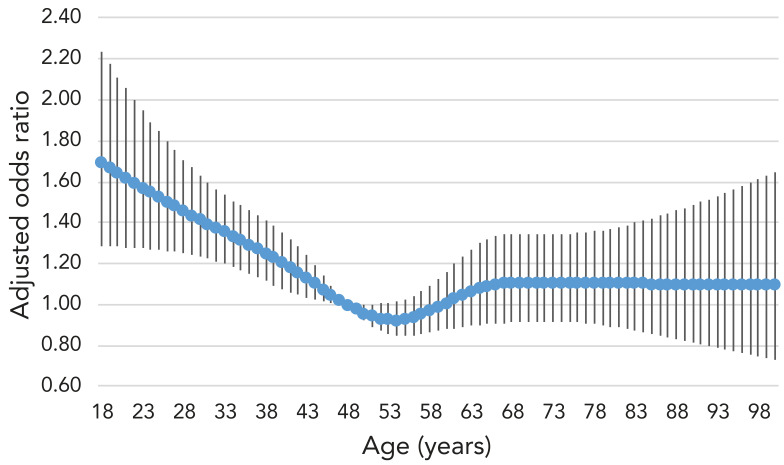
Adjusted odds ratio for oral antibiotic use in the past 12 months by age

The mean age of respondents was 48.1 years old, which served as the reference for the logistic regression models. After adjusting for all other exposure variables, those aged 18 years had much higher odds, 1.70 (95% CI: 1.29–2.23) compared to those aged 48 years (**Table 2**). Adults aged 30 years had odds of 1.42 (95% CI: 1.23–1.63); at age 60, the odds were 1.01 (95% CI: 0.88–1.16) and at age 80, the odds were 1.11 (95% CI: 0.89–1.37) compared to those aged 48 years (see **Figure 1** for unadjusted odds and **Figure 2** for adjusted odds).

**Table 2:** Characteristics associated with receiving an antibiotic prescription in the previous 12 months

Characteristics	Unweighted		*p*-value		Odds ratio	
	Frequency	Percent (%)			Unadjusted	Adjusted
**Age (years)**						
**Mean (SEM)**	**48.11 (48.0–48.22)**		**<0.0001**		**See ** **Figure 1** ** and ** **Figure 2**	
18–29	5,472,681 (5,303,207–5,642,156)	18.86 (18.27–19.44)	-		Not included in model, age was treated as continuous	
30–39	5,255,468 (5,017,771–5,493,165)	18.11 (17.29–18.93)				
40–49	4,668,792 (4,508,772–4,828,812)	16.09 (15.54–16.64)				
50–59	5,013,909 (4,856,837–5,170,981)	17.28 (16.74–17.82)				
60–69	4,698,262 (4,497,236–4,899,288)	16.19 (15.5–16.88)				
70–79	2,693,963 (2,572,919–2,815,007)	9.28 (8.87–9.7)				
80+	1,217,479 (1,125,974–1,308,983)	4.2 (3.88–4.51)				
**Sex**				**<0.0001**		
Female	14,742,425 (14,742,424–14,742,426)	50.8 (50.8–50.8)	-		1.65 (1.49–1.83)	1.55 (1.38–1.72)
Male	14,278,128 (14,278,127–14,278,128)	49.2 (49.2–49.2)			Ref.	
**Highest level of education**				**0.0029**		
High school	10,333,492 (9,999,596–10,667,387)	35.61 (34.46–36.76)	-		0.91 (0.79–1.05)	0.77 (0.66–0.89)
Diploma	10,371,261 (10,058,907–10,683,616)	35.74 (34.66–36.82)			0.95 (0.84–1.07)	0.88 (0.77–1.01)
University	8,315,800 (7,987,390–8,644,210)	28.65 (27.52–29.79)			Ref.	
**Smoking status**				**0.0063**		
Current	4,872,020 (4,617,655–5,126,385)	16.79 (15.91–17.67)	-		1.31 (1.13–1.51)	1.3 (1.11–1.53)
Experiment	3,914,117 (3,696,472–4,131,761)	13.49 (12.74–14.24)			1.11 (0.94–1.29)	1.14 (0.97–1.34)
Former	7,704,652 (7,421,722–7,987,581)	26.55 (25.57–27.53)			1.2 (1.06–1.36)	1.22 (1.06–1.4)
Never	12,529,764 (12,185,432–12,874,096)	43.18 (41.99–44.36)			Ref.	
**Indigenous (off-reserve)/immigrant status**				**0.1067**		
Indigenous (off-reserve)	978,508 (870,556–1,086,460)	3.37 (3.0–3.74)	-		1.2 (0.94–1.53)	1.04 (0.81–1.34)
Immigrant	7,492,618 (7,126,684–7,858,551)	25.82 (24.56–27.08)	-		0.94 (0.82–1.08)	1.21 (1.01–1.45)
Non-Indigenous/non-immigrant	20,549,427 (20,193,939–20,904,9150	70.81 (69.58–72.04)	-		Ref.	
**Total household income (thousands)**				**0.7555**		
<50	7,588,111 (7,288,500–7,887,721)	26.15 (25.11–27.18)	-		1.11 (0.93–1.31)	0.94 (0.78–1.14)
50–100	9,303,183 (9,011,645–9,594,722)	32.06 (31.05–33.06)			0.99 (0.84–1.16)	0.92 (0.78–1.09)
100–149	6,033,084 (5,772,751–6,293,418)	20.79 (19.89–21.69)			0.97 (0.82–1.15)	0.92 (0.77–1.09)
>150	6,096,174 (5,804,118–6,388,231)	21.01 (20.0–22.01)			Ref.	
**Perceived health**				**<0.0001**		
Poor/fair	3,487,551 (3,276,377–3,698,725)	12.02 (11.29–12.75)	-		2.82 (2.36–3.38)	1.89 (1.45–2.46)
Good	8,341,719 (8,033,243–8,650,196)	28.74 (27.68–29.81)			1.75 (1.51–2.04)	1.47 (1.22–1.75)
Very good	10,588,084 (10,279,864–10,896,303)	36.48 (35.42–37.55)			1.47 (1.27–1.7)	1.34 (1.14–1.57)
Excellent	6,603,198 (6,323,648–6,882,749)	22.75 (21.79–23.72)			Ref.	
**Perceived life stress**				**0.0003**		
Not at all stressful	3,957,912 (3,760,455–4,155,369)	13.64 (12.96–14.32)	-		Ref.	
Not very stressful	6,783,011 (6,515,591–7,050,432)	23.37 (22.45–24.3)			1.35 (1.13–1.61)	1.29 (1.07–1.55)
A bit stressful	11,999,017 (11,679,691–12,318,343)	41.35 (40.24–42.45)			1.61 (1.36–1.91)	1.42 (1.18–1.72)
Stressful	6,280,612 (5,995,955–6,565,269)	21.64 (20.66–22.62)			2.05 (1.68–2.51)	1.62 (1.29–2.04)
**Chronic medical condition(s)**						
Has asthma	2,413,833 (2,237,478–2,590,188)	8.32 (7.71–8.93)	0.0001		1.88 (1.58–2.24)	1.44 (1.2–1.74)
Has chronic obstructive pulmonary disease	838,936 (743,407–934,466)	2.89 (2.56–3.22)	<0.0001		2.83 (2.23–3.59)	1.92 (1.45–2.53)
Has arthritis	5,790,867 (5,564,474–6,017,260)	19.95 (19.17–20.74)	0.0001		1.57 (1.4–1.75)	1.29 (1.13–1.47)
Has high blood pressure	5,326,295 (5,092,116–5,560,473)	18.35 (17.55–19.16)	0.0249		1.08 (0.96–1.21)	0.85 (0.74–0.98)
Has high blood cholesterol/lipids	3,686,570 (3,491,111–5,560,473)	12.7 (12.03–13.38)	0.4780		1.21 (1.06–1.39)	1.06 (0.9–1.25)
Has heart disease	1,382,509 (1,248,851–1,516,167)	4.76 (4.3–5.23)	0.0004		1.72 (1.43–2.07)	1.45 (1.18–1.79)
Ever been diagnosed with cancer	2,175,846 (2,030,344–2,321,349)	7.5 (7.0–8.0)	0.0157		1.41 (1.22–1.62)	1.23 (1.04–1.46)
Has a bowel disorder (Crohn's disease, ulcerative colitis, irritable bowel syndrome, incontinence)	1,558,896 (1,431,507–1,686,285)	5.37 (4.93–5.81)	0.0080		1.91 (1.63–2.24)	1.27 (1.07–1.52)
Has urinary incontinence	1,146,488 (1,028,631–1,265,228)	3.95 (3.54–4.36)	0.0265		1.85 (1.5–2.27)	1.31 (1.03–1.67)
**Usual place for immediate care for minor problems**				**<0.0001**		
Community health centre	1,146,488 (1,030,584–1,262,392)	3.95 (3.55–4.35)	-		0.77 (0.6–0.98)	0.78 (0.6–1.02)
Doctor's office	14,534,280 (14,210,494–14,858,067)	50.08 (48.97–51.2)			Ref.	
Emergency room	1,944,944 (1,792,576–2,097,311)	6.7 (6.18–7.23)			0.91 (0.74–1.11)	1 (0.8–1.23)
Hospital outpatient	725,183 (642,555–807,811)	2.5 (2.21–2.78)			0.82 (0.64–1.06)	0.83 (0.63–1.08)
Walk-in clinic	6,889,707 (6,603,742–7,175,672)	23.74 (22.75–24.73)			0.99 (0.87–1.13)	1.1 (0.95–1.26)
No usual place of care	3,779,951 (3,566,350–3,993,551)	13.03 (12.29–13.76)			0.56 (0.46–0.66)	0.66 (0.54–0.8)
**Regular provider type**				**0.0004**		
FP/GP	23,941,588 (23,683,672–24,199,503)	82.5 (81.61–83.39)	-		Ref.	
Non-FP/GP	732,110 (605,057–859,163)	2.52 (2.08–2.96)			0.87 (0.62–1.23)	0.84 (0.58–1.23)
No usual provider	4,346,855 (4,123,637–4,570,073)	14.98 (14.21–15.75)			0.61 (0.53–0.71)	0.71 (0.6–0.84)

Abbreviations: FP, family practitioner; GP, general practitioner; Ref., reference; SEM, standard error of the mean

Note: These additional covariates were also included in the model: province of residence, marital status, body mass index, type of drinker, physical activity, insurance for prescription medications, language most often spoken at home, perceived mental health, seasonal flu shot, stroke, diabetes, mood disorder and anxiety disorder. Adjusted and unadjusted results for these covariates can be found in the Appendix

In the adjusted model, women had higher odds of reporting receipt of an antibiotic prescription in the previous 12 months compared to men (OR 1.55; 95% CI: 1.38–1.72) (Table 2). Using the adjusted logistic regression model, immigrants were 1.21 (95% CI: 1.01–1.45) times more likely than those who were both non-Indigenous and non-immigrants to report receiving an antibiotic prescription. For Indigenous respondents (off-reserve), the odds were 1.04 (95% CI: 0.81–1.34) times higher, however, it was not possible to determine if this difference was significant due to the small number of Indigenous respondents (3.37%).

Respondents who reported having no usual place of care for minor medical problems (OR 0.66; 95% CI: 0.54–0.80) or no regular healthcare provider (OR 0.71; 95% CI: 0.60–0.84) were less likely to receive an antibiotic prescription after adjusting for all other covariates (Table 2).

Those who self-reported less than excellent health and perceived life stress had greater odds of receiving an antibiotic prescription. Both current and former smokers had higher odds compared to those who had never smoked. Asthma, chronic obstructive pulmonary disease, arthritis, heart disease, cancer, bowel disorders and urinary incontinence were associated with an increased odds of receiving a prescription. Hypertension was associated with lower odds. The frequency of responses was too low to include receipt of seasonal influenza vaccination in the model.

## Discussion

This study revealed that about one-quarter of Canadians (26.0%) received at least one systemic (oral) antibiotic prescription over a one-year period, of whom 38% received more than one. One in five of these prescriptions (21.5%) was reported to be for a chest infection. This is concerning given that bronchitis has been found to be associated with high levels of unnecessary antibiotic prescribing in other research (52% in British Columbia ((11)); 53% in Ontario) ((5)). The high proportion of reported prescriptions for ear/nose/throat/sinus/eye infections (23.2%) is similarly notable, given that previous research has found a high rate of unnecessary prescribing for sinus infections (48% in British Columbia; 48% in Ontario), throat infections (42% in British Columbia) and ear infections (39% in Ontario) ((5,11)).

After controlling for medical conditions, the odds of those aged 18 years and those aged 30 years having received a prescription were higher than those aged 48 years, 60 years and 80 years. It is expected for antibiotic use to rise with age and for much of it to be attributable to greater morbidity, however, it is unclear what underpins young adults' odds of use such that it surpasses the odds for middle-age and older adults when controlling for medical conditions. Younger adults may be more likely to have a faulty understanding of what constitutes an oral antibiotic. As well, this survey does not capture the frailest older adults, such as long-term care residents or those in hospital, possibly eliminating a large portion of antibiotic use in these disproportionately elderly groups. Population usage metrics show a greater burden of antibiotic use among older age groups ((2)). Taken together, these different measures might also indicate that those older adults who use antibiotics use a high quantity (by tonnage or by prescription) while young adults may have more evenly distributed use across their age groups or shorter prescriptions. These findings are similar to those of other surveys on antibiotic use in Canada that found high reported use among young adults ((12,13)). Younger age groups also have a much higher burden of conditions that are frequently treated with antibiotics that were not controlled for in our study, such as sexually transmitted infections ((14)) and acne ((15)). The widespread and intensive use of systemic antibiotics for acne, particularly among young adults, has notably been challenged in recent scientific literature and guidelines have been changed in many regions to reduce their use to limit AMR ((16−19)). Young adults may also be parents and are more likely to be exposed to respiratory infections through their children ((20,21)). In some contexts, young adults have a higher rate of inappropriate prescriptions for upper respiratory tract infections than other adult age groups ((22,23)).

In line with previously published findings in the literature and Canadian dispensation data ((2,8)), antibiotic use is higher among women. This may be for reasons linked to biology (e.g., a higher risk of urinary tract infections) or gendered social dynamics (e.g., a higher likelihood to seek medical care ((24)) and very high representation in work with exposure to patients, children or food-labour sectors associated with higher rates of infections ((25))).

Contrary to other studies from high-income countries, neither income nor education were significant in either adjusted or unadjusted analyses ((8)). This may be because we were able to control for other variables that are often co-linear with socioeconomic status such as comorbidities (positively associated with use) and low levels of access to regular medical care (negatively associated with use).

We found very slightly higher use among Indigenous populations off-reserve. This contrasts with other studies that have found high dispensation rates of antibiotics to Indigenous populations on-reserve and in the Arctic ((26,27)). However, it is in line with studies that have found that antibiotic use is not highly different in regions with higher Indigenous populations, though the latter studies also appear to have excluded on-reserve dispensations, potentially skewing regional use and its associations ((28,29)).

The finding of higher use among immigrant populations in Canada departs from a study that found that regions in Ontario with a higher proportion of immigrants had neither higher nor lower use ((28)).

In accordance with many other findings, several medical conditions were associated with higher antibiotic use, which is potentially explained by the need for invasive devices with elevated risk of infection, depressed immunity, symptoms of unclear etiology or frequent interactions with medical care. The finding that hypertension was associated with lower odds of prescriptions may be explained by known contraindications of blood pressure medications with use of certain antibiotics ((30,31)).

### Limitations

The results are based on self-reported survey data, and responses may reflect recall bias or social desirability bias. Respondents may also have a faulty understanding of what an antibiotic is. This is a common and well-known limitation in surveys of antibiotic use ((32−35)). While restricting participation to respondents who demonstrate knowledge of antibiotic use could mitigate this issue, it would introduce selection bias ((32)).

These results do not include the Territories or residents of the remote Québec regions of Nunavik and Terres-Cries-de-la-Baie-James, Indigenous communities, institutionalized populations (e.g., residents of healthcare facilities, long-term care, prisons, convents) and full-time members of the Canadian Armed Forces. This survey does not include unprescribed antibiotic use, which in other contexts has been found to be higher among certain demographics, including migrant workers, men who have sex with men and people who inject drugs ((22,36)). Additionally, telephone surveys may not capture the frailest community-dwelling adults and will not capture people without a phone, which may both be key populations for high antibiotic use ((8)). As well, recent research has highlighted very elevated levels of antibiotic prescribing to gay, bisexual, and other men who have sex with men in an urban sexual health clinic ((37)), to people living in Arctic communities ((27)) and to First Nations individuals accessing health care at nursing stations on-reserve in Canada ((26)). Further research should further inquire into levels of AMU among these populations at a national level.

## Conclusion

These results suggest that efforts to reduce unnecessary antibiotic use through stewardship and policy initiatives need to target the whole age spectrum. More data are necessary to understand and address the drivers of antibiotic use and to elucidate why young people have higher odds of being prescribed an antibiotic than those in middle-age when controlling for other factors, similar to what has been seen in other studies ((12,13)). Medical record data may help elucidate why certain comorbidities are associated with higher antibiotic use and help capture if it is appropriate or not to better tailor stewardship interventions.

In order to best tailor interventions on antibiotic use for immigrant communities, further research is necessary to identify which ethnocultural and linguistic groups are most affected. As well, more research and better data are needed on key populations not included in this study of AMU, including Indigenous people on-reserve and in the Arctic, individuals in long-term care establishments, two-spirit, gay, and bisexual men who have sex with men, transgender populations, incarcerated populations and people who use drugs, particularly by injection.

Notably, just over a quarter of respondents reported having taken systemic oral antibiotics, most frequently for indications for which close to half of prescriptions are known to be inappropriate. This points to the need for better education of prescribers and Canadians on the role of judicious AMU in protecting individual health and the health of the community.

## Appendix

**Table A1:** Characteristics associated with receiving an antibiotic prescription in the previous 12 months with all variables

Characteristics	Weighted		Odds ratio		
	Frequency	Percent (%)	Unadjusted	Adjusted	
**Age (years)**					
**Mean (SEM)**	**48.11 (48.0–48.22)**		**See ** **Figure 1**		
18–29	5,472,681 (5,303,207–5,642,156)	18.86 (18.27–19.44)	Not included in model, age was treated as continuous		
30–39	5,255,468 (5,017,771–5,493,165)	18.11 (17.29–18.93)			
40–49	4,668,792 (4,508,772–4,828,812)	16.09 (15.54–16.64)			
50–59	5,013,909 (4,856,837–5,170,981)	17.28 (16.74–17.82)			
60–69	4,698,262 (4,497,236–4,899,288)	16.19 (15.5–16.88)			
70–79	2,693,963 (2,572,919–2,815,007)	9.28 (8.87–9.7)			
80+	1,217,479 (1,125,974–1,308,983)	4.2 (3.88–4.51)			
**Sex**					
Female	14,742,425 (14,742,424–14,742,426)	50.8 (50.8–50.8)	1.65 (1.49–1.83)		1.55 (1.38–1.72)
Male	14,278,128 (14,278,127–14,278,128)	49.2 (49.2–49.2)	Ref.		
**Marital status**					
Married/common-law	18,199,194 (17,888,721–18,509,667)	62.71 (61.64–63.78)	Ref.		
Single	7,070,640 (6,828,042–7,313,238)	24.36 (23.53–25.2)	1.03 (0.89–1.18)		0.95 (0.8–1.14)
Widowed/separated/divorced	3,750,719 (3,567,739–3,933,700)	12.92 (12.29–13.56)	1.11 (0.97–1.26)		0.94 (0.8–1.09)
**Highest level of education**					
High school	10,333,492 (9,999,596–10,667,387)	35.61 (34.46–36.76)	0.91 (0.79–1.05)		0.77 (0.66–0.89)
Diploma	10,371,261 (10,058,907–10,683,616)	35.74 (34.66–36.82)	0.95 (0.84–1.07)		0.88 (0.77–1.01)
University	8,315,800 (7,987,390–8,644,210)	28.65 (27.52–29.79)	Ref.		
**Body mass index**					
Underweight	420,444 (330,287–510,601)	1.45 (1.14–1.76)	0.98 (0.6–1.61)		0.79 (0.46–1.33)
Normal weight	9,389,187 (9,068,169–9,710,205)	32.35 (31.25–33.46)	Ref.		
Overweight	10,036,834 (9,720,884–10,352,785)	34.59 (33.5–35.68)	0.91 (0.8–1.03)		0.97 (0.84–1.11)
Obese I	4,724,310 (4,496,430–4,952,190)	16.28 (15.49–17.07)	1.07 (0.92–1.23)		1.03 (0.88–1.21)
Obese II	1,645,989 (1,503,982–1,787,997)	5.67 (5.18–6.16)	1.38 (1.13–1.69)		1.24 (1.0–1.54)
Obese III	897,816 (788,814–1,006,819)	3.09 (2.72–3.47)	1.49 (1.14–1.95)		1.18 (0.89–1.56)
Unknown	1,905,972 (1,730,180–2,081,763)	6.57 (5.96–7.17)	0.99 (0.79–1.23)		0.84 (0.66–1.07)
**Smoking status**					
Current	4,872,020 (4,617,655–5,126,385)	16.79 (15.91–17.67)	1.31 (1.13–1.51)		1.3 (1.11–1.53)
Experiment	3,914,117 (3,696,472–4,131,761)	13.49 (12.74–14.24)	1.11 (0.94–1.29)		1.14 (0.97–1.34)
Former	7,704,652 (7,421,722–7,987,581)	26.55 (25.57–27.53)	1.2 (1.06–1.36)		1.22 (1.06–1.4)
Never	12,529,764 (12,185,432–12,874,096)	43.18 (41.99–44.36)	Ref.		
**Type of drinker (last 12 months)**					
Never	6,098,171 (5,786,026–6,410,317)	21.01 (19.94–22.09)	Ref.		
Occasional	4,861,665 (4,609,049–5,114,282)	16.75 (15.88–17.62)	1.04 (0.88–1.22)		1.0 (0.84–1.19)
Regular	18,060,716 (17,708,394–18,413,038)	62.23 (61.02–63.45)	0.93 (0.81–1.06)		1.0 (0.86–1.15)
**Physical activity**					
Active	10,675,829 (10,346,693–11,004,965)	36.79 (35.65–37.92)	Ref.		
Moderately active	4,926,604 (4,685,618–5,167,590)	16.98 (16.14–17.81)	1.15 (0.99–1.34)		1.08 (0.93–1.26)
Somewhat active	6,472,529 (6,191,847–6,753,211)	22.3 (21.33–23.27)	1.14 (1.0–1.31)		1.05 (0.91–1.21)
Sedentary	6,945,590 (6,672,435–7,218,745)	23.93 (22.99–24.88)	1.14 (1.0–1.3)		1.0 (0.86–1.16)
**Indigenous (off-reserve)/immigrant status**					
Indigenous (off-reserve)	978,508 (870,556–1,086,460)	3.37 (3.0–3.74)	1.2 (0.94–1.53)		1.04 (0.81–1.34)
Immigrant	7,492,618 (7,126,684–7,858,551)	25.82 (24.56–27.08)	0.94 (0.82–1.08)		1.21 (1.01–1.45)
Non-Indigenous/non-immigrant	20,549,427 (20,193,939–20,904,915)	70.81 (69.58–72.04)	Ref.		
**Language most often spoken at home (first answer)**					
English	18,759,089 (18,414,234–19,103,944)	64.64 (63.45–65.83)	Ref.		
French	5,915,950 (5,767,569–6,064,331)	20.39 (19.87–20.9)	0.97 (0.87–1.08)		1.05 (0.82–1.35)
Other	4,345,514 (3,998,878–4,692,150)	14.97 (13.78–16.17)	0.82 (0.69–0.98)		0.89 (0.7–1.12)
**Total household income (thousands)**					
<50	7,588,111 (7,288,500–7,887,721)	26.15 (25.11–27.18)	1.11 (0.93–1.31)		0.94 (0.78–1.14)
50–100	9,303,183 (9,011,645–9,594,722)	32.06 (31.05–33.06)	0.99 (0.84–1.16)		0.92 (0.78–1.09)
100–149	6,033,084 (5,772,751–6,293,418)	20.79 (19.89–21.69)	0.97 (0.82–1.15)		0.92 (0.77–1.09)
>150	6,096,174 (5,804,118–6,388,231)	21.01 (20.0–22.01)	Ref.		
**Province of residence**					
Alberta	3,319,229 (3,319,228–3,319,229)	11.44 (11.44–11.44)	1.02 (0.87–1.2)		1.05 (0.89–1.24)
British Columbia	3,867,378 (3,867,377–3,867,378)	13.33 (13.33–13.33)	1.02 (0.87–1.19)		1.07 (0.9–1.27)
Manitoba	977,254 (977,254–977,254)	3.37 (3.37–3.37)	0.99 (0.8–1.22)		1.11 (0.88–1.39)
New Brunswick	603,559 (603,559–603,560)	2.08 (2.08–2.08)	1.1 (0.87–1.38)		1.12 (0.87–1.44)
Newfoundland and Labrador	428,946 (428,946–428,947)	1.48 (1.48–1.48)	1.27 (1.0–1.6)		1.42 (1.1–1.84)
Nova Scotia	768,501 (768,501–768,501)	2.65 (2.65–2.65)	1.19 (0.98–1.44)		1.17 (0.94–1.45)
Ontario	11,377,324 (11,377,324–11,377,324)	39.2 (39.2–39.2)	Ref.		
Prince Edward Island	120,209 (120,209–120,209)	0.41 (0.41–0.41)	1.29 (1.0–1.67)		1.39 (1.04–1.86)
Québec	6,712,348 (6,712,347–6,712,348)	23.13 (23.13–23.13)	0.99 (0.87–1.13)		1.1 (0.86–1.42)
Saskatchewan	845,805 (845,805–845,805)	2.91 (2.91–2.91)	1.07 (0.87–1.33)		1.12 (0.89–1.41)
**Perceived health**					
Poor/fair	3,487,551 (3,276,377–3,698,725)	12.02 (11.29–12.75)	2.82 (2.36–3.38)		1.89 (1.45–2.46)
Good	8,341,719 (8,033,243–8,650,196)	28.74 (27.68–29.81)	1.75 (1.51–2.04)		1.47 (1.22–1.75)
Very good	10,588,084 (10,279,864–10,896,303)	36.48 (35.42–37.55)	1.47 (1.27–1.7)		1.34 (1.14–1.57)
Excellent	6,603,198 (6,323,648–6,882,749)	22.75 (21.79–23.72)	Ref.		
**Perceived mental health**					
Poor/fair	2,103,157 (1,926,551–2,279,763)	7.25 (6.64–7.86)	2.01 (1.64–2.48)		1.03 (0.79–1.33)
Good	7,680,865 (7,390,689–7,971,041)	26.47 (25.47–27.47)	1.45 (1.26–1.67)		1.07 (0.91–1.26)
Very good	10,430,576 (10,111,131–10,750,020)	35.94 (34.84–37.04)	1.08 (0.95–1.22)		0.9 (0.78–1.03)
Excellent	8,805,955 (8,505,460–9,106,450)	30.34 (29.31–31.38)	Ref.		
**Perceived life stress**					
Not at all stressful	3,957,912 (3,760,455–4,155,369)	13.64 (12.96–14.32)	Ref.		
Not very stressful	6,783,011 (6,515,591–7,050,432)	23.37 (22.45–24.3)	1.35 (1.13–1.61)		1.29 (1.07–1.55)
A bit stressful	11,999,017 (11,679,691–12,318,343)	41.35 (40.24–42.45)	1.61 (1.36–1.91)		1.42 (1.18–1.72)
Stressful	6,280,612 (5,995,955–6,565,269)	21.64 (20.66–22.62)	2.05 (1.68–2.51)		1.62 (1.29–2.04)
Had a seasonal flu shot (current/last year)	56,010 (36,132–75,888)	0.19 (0.12–0.26)	Frequency too low to include in model		
**Chronic medical condition(s)**					
Has asthma	2,413,833 (2,237,478–2,590,188)	8.32 (7.71–8.93)	1.88 (1.58–2.24)		1.44 (1.2–1.74)
Has chronic obstructive pulmonary disease	838,936 (743,407–934,466)	2.89 (2.56–3.22)	2.83 (2.23–3.59)		1.92 (1.45–2.53)
Has arthritis	5,790,867 (5,564,474–6,017,260)	19.95 (19.17–20.74)	1.57 (1.4–1.75)		1.29 (1.13–1.47)
Has high blood pressure	5,326,295 (5,092,116–5,560,473)	18.35 (17.55–19.16)	1.08 (0.96–1.21)		0.85 (0.74–0.98)
Has high blood cholesterol/lipids	3,686,570 (3,491,111–3,882,029)	12.7 (12.03–13.38)	1.21 (1.06–1.39)		1.06 (0.9–1.25)
Has heart disease	1,382,509 (1,248,851–1,516,167)	4.76 (4.3–5.23)	1.72 (1.43–2.07)		1.45 (1.18–1.79)
Suffers from the effects of a stroke	376,726 (310,763–442,688)	1.3 (1.07–1.53)	1.36 (0.95–1.94)		0.88 (0.58–1.33)
Has diabetes	2,221,519 (2,062,356–2,380,683)	7.65 (7.11–8.2)	1.3 (1.11–1.53)		1.08 (0.9–1.29)
Ever been diagnosed with cancer	2,175,846 (2,030,344–2,321,349)	7.5 (7.0–8.0)	1.41 (1.22–1.62)		1.23 (1.04–1.46)
Has a bowel disorder (Crohn's disease, ulcerative colitis, irritable bowel syndrome, incontinence)	1,558,896 (1,431,507–1,686,285)	5.37 (4.93–5.81)	1.91 (1.63–2.24)		1.27 (1.07–1.52)
Has urinary incontinence	1,146,930 (1,028,631–1,265,228)	3.95 (3.54–4.36)	1.85 (1.5–2.27)		1.31 (1.03–1.67)
Has a mood disorder (depression, bipolar, mania, dysthmia)	2,620,823 (2,425,021–2,816,626)	9.03 (8.36–9.71)	1.85 (1.57–2.18)		1.15 (0.93–1.42)
Has an anxiety disorder (phobia, obsessive compulsive disorder, panic disorder)	2,615,767 (2,424,766–2,806,769)	9.01 (8.35–9.67)	1.68 (1.44–1.97)		1.02 (0.86–1.22)
**Usual place for immediate care for minor problems**					
Community health centre	1,146,488 (1,030,584–1,262,392)	3.95 (3.55–4.35)	0.77 (0.6–0.98)		0.78 (0.6–1.02)
Doctor's office	14,534,280 (14,210,494–14,858,067)	50.08 (48.97–51.2)	Ref.		
Emergency room	1,944,944 (1,792,576–2,097,311)	6.7 (6.18–7.23)	0.91 (0.74–1.11)		1.0 (0.8–1.23)
Hospital outpatient	725,183 (642,555–807,811)	2.5 (2.21–2.78)	0.82 (0.64–1.06)		0.83 (0.63–1.08)
Walk-in clinic	6,889,707 (6,603,742–7,175,672)	23.74 (22.75–24.73)	0.99 (0.87–1.13)		1.1 (0.95–1.26)
No usual place of care	3,779,951 (3,566,350–3,993,551)	13.03 (12.29–13.76)	0.56 (0.46–0.66)		0.66 (0.54–0.8)
**Regular provider type**					
FP/GP	23,941,588 (23,683,672–24,199,503)	82.5 (81.61–83.39)	Ref.		
Non-FP/GP	732,110 (605,057–859,163)	2.52 (2.08–2.96)	0.87 (0.62–1.23)		0.84 (0.58–1.23)
No usual provider	4,346,855 (4,123,637–4,570,073)	14.98 (14.21–15.75)	0.61 (0.53–0.71)		0.71 (0.6–0.84)
Insurance for prescription medications (all or part coverage)	22,877,375 (22,616,076–23,138,674)	78.83 (77.93–79.73)	1.27 (1.11–1.45)		1.15 (1.0–1.32)

Abbreviations: FP, family practitioner; GP, general practitioner; Ref., reference; SEM, standard error of the mean
